# Approved Small-Molecule ATP-Competitive Kinases Drugs Containing Indole/Azaindole/Oxindole Scaffolds: R&D and Binding Patterns Profiling

**DOI:** 10.3390/molecules28030943

**Published:** 2023-01-17

**Authors:** Haofan Zhang, Fengming He, Guiping Gao, Sheng Lu, Qiaochu Wei, Hongyu Hu, Zhen Wu, Meijuan Fang, Xiumin Wang

**Affiliations:** 1 School of Pharmaceutical Sciences, Xiamen University, Xiamen 361102, China; 2 School of Medicine, Huaqiao University, Quanzhou 362021, China; 3 School of Public Health, Xiamen University, Xiamen 361102, China; 4 Xingzhi College, Zhejiang Normal University, Lanxi 321004, China

**Keywords:** indole/azaindole/oxindole derivatives, approved ATP-competitive kinases drugs, cancer, drug design, kinases binding patterns, drug discovery

## Abstract

Kinases are among the most important families of biomolecules and play an essential role in the regulation of cell proliferation, apoptosis, metabolism, and other critical physiological processes. The dysregulation and gene mutation of kinases are linked to the occurrence and development of various human diseases, especially cancer. As a result, a growing number of small-molecule drugs based on kinase targets are being successfully developed and approved for the treatment of many diseases. The indole/azaindole/oxindole moieties are important key pharmacophores of many bioactive compounds and are generally used as excellent scaffolds for drug discovery in medicinal chemistry. To date, 30 ATP-competitive kinase inhibitors bearing the indole/azaindole/oxindole scaffold have been approved for the treatment of diseases. Herein, we summarize their research and development (R&D) process and describe their binding models to the ATP-binding sites of the target kinases. Moreover, we discuss the significant role of the indole/azaindole/oxindole skeletons in the interaction of their parent drug and target kinases, providing new medicinal chemistry inspiration and ideas for the subsequent development and optimization of kinase inhibitors.

## 1. Introduction

Kinases are a class of biochemical molecules that can transfer phosphate groups from high-energy donor molecules, such as ATP, to specific target molecules (substrates), a process called phosphorylation that ultimately leads to the altered biological function of the target protein [1]. Abnormal expression of many kinases leads to inflammatory diseases [2], metabolic diseases [3], cancer [4], etc. Therefore, kinases are extremely significant targets for drug development.

The structure of kinase was first described by Knighton et al. in 1991 [4]. The conserved structure of the catalytic structural domain of kinase consists mainly of N-terminal and C-terminal lobes connected by a short loop in the hinge region [5,6]. The N-lobe flap consists of five antiparallel β-strands and one Cα-helix, whereas the C-terminal flap consists of eight α-helices and four β-strands. The region between the N-terminal and C-terminal lobes and the hinge region forms a deep hydrophobic cleft containing the ATP-binding site. The adenine group of ATP forms several critical hydrogen bond interactions with the backbone atoms of the hinge residues, thereby facilitating the stable binding of ATP within the catalytic pocket. In addition, the phosphate binding loop forms the ceiling of the ATP binding site and clamps on the phosphate group to allow for targeted catalysis. The protein substrate binding site is located within the C-lobe, where the activation loop is also located in the C-terminal lobe. Many kinases are phosphorylated within this loop and then undergo conformational changes to activate the kinase and allow access to the substrate binding site. In addition to the catalytic structural domain, kinases may also contain other regulatory structural domains, and ATP is also coordinated by a glycine-rich ring (G-loop), a highly flexible region that is present in the β-sheet structures. These regulatory structural domains play different roles in the kinome, including regulating catalytic activity, recruiting substrates, controlling localization, and acting as scaffolding sites for other proteins [7,8,9]. The FDA approved Imatinib in 2001 as the first marketed targeted tyrosine kinase (ABL) inhibitor for the treatment of chronic myeloid leukemia (CML), which was a breakthrough in molecularly targeted cancer therapy [10]. Since then, research on kinase-related signaling pathways and the development of related targeted drugs have been proceeding promptly; until 2021, 87 small-molecule kinase inhibitors have been approved for the treatment of a variety of diseases, including tumors [11,12].

In order to better understand the mechanism of action of small-molecule kinases inhibitors, researchers have classified these inhibitors into four types based on their binding models. Type I inhibitors, which typically consist of a heterocyclic system occupying an adenine binding site and side chains occupying adjacent hydrophobic regions, target the ATP pocket of the kinase in its active state. Type II inhibitors bind to the ATP-binding site and an additional back hydrophobic pocket in the inactive state. Type III inhibitors are allosteric kinase inhibitors that bind to allosteric sites in the vicinity of the ATP site that do not compete with ATP. Type IV inhibitors are also allosteric kinase inhibitors that bind to allosteric sites in the structural domain of the kinase away from the ATP pocket [12,13,14]. Type III and IV inhibitors are classified primarily based on the distance of small-molecule allosteric kinase inhibitors from the ATP-binding pocket to the allosteric site [15].

This perspective focuses on ATP-competitive kinase inhibitors (type I and type II), and it is found that indole/azaindole/oxindole parent cores appear in numerous ATP-competitive kinase inhibitors [16,17,18]. This may be due to the fact that indole/azaindole/oxindole-containing fragments can better fit Lipinski’s rule than other bicyclic fused heterocycles. The indole/azaindole/oxindole scaffold competitively occupies the ATP-binding pocket of the kinase and interacts with key residues in the binding site with non-covalent bonding interactions such as hinge hydrogen bonds, pi–pi stacking, pi–cation, etc., thereby inhibiting the kinase activity and thus regulating kinase-related signaling pathways. This paper provides a detailed review of the discovery of thirty approved, three in clinical trials, and one in preclinical studied ATP-competitive small-molecule kinase inhibitors containing indoles/azaindole/oxindole scaffolds and their binding patterns to the ATP-binding pockets of their target kinases. The profiling of these binding models based on X-ray crystal structures provides structural insights for the design of more desirable and selective ATP-competitive small-molecule inhibitors. All of the 3D diagrams of the binding model analyses were rendered by PyMOL (The PyMOL Molecular Graphics System, Version 2.3 Schrödinger, LLC, New York, NY, USA). Among them, the violet dashed lines denote hydrogen bonds, the orange dashed lines represent salt bridges, the red dashed lines represent pi–cation interactions, the green dashed lines indicate pi–pi stacking, key residues that have non-covalent interactions with ligands are represented in yellow sticks, and proteins are shown as light red cartoons. In addition, all 2D interaction illustrations were generated using PoseView [19,20], where the hinge hydrogen bonds are highlighted in violet, and the hinge residues and the indole/azaindole/oxindole skeletons are highlighted in yellow.

## 2. Indole/Azaindole/Oxindole-Based Approved ATP-Competitive Kinase Drugs

### 2.1. The Breakpoint Cluster Region Abelson (Bcr-Abl) Inhibitors

The breakpoint cluster region Abelson (Bcr-Abl) tyrosine kinase (TK) is a constitutively activated cytoplasmic TK that is an underlying cause of CML [21]. In 2001, the Bcr-Abl inhibitor Imatinib was used as first-line therapy for the treatment of CML [18]. However, clinical findings suggest that patients may develop drug resistance, especially in the advanced stages of the disease [22,23,24]. Mutations in the structural domain of the kinase are the main reason for Bcr-Abl inhibitor resistance, and one of the most common mutations in Bcr-Abl is the substitution of threonine 315 (located in the center of the Imatinib binding site) by isoleucine [25,26,27]. The drug resistance problem has prompted researchers to search for lines of Bcr-Abl inhibitors.

**Figure 1:**
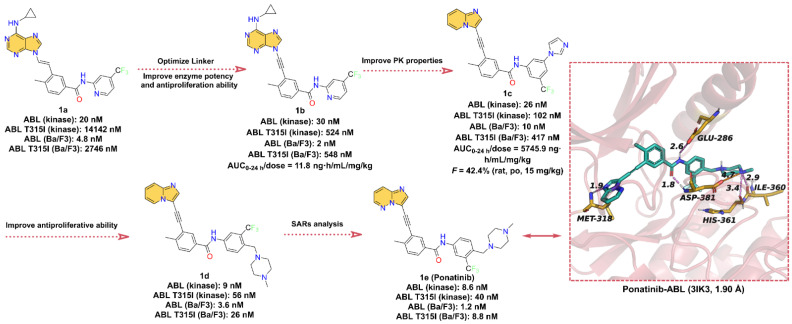
Key medicinal chemistry optimization leading to **Ponatinib** and its binding model with Bcr-Abl.

In this section, we concentrate on ATP-competitive inhibitors that target Bcr-Abl and contain azaindole scaffold (Figure 1). **1e** (Ponatinib) was approved for second-line treatment of chronic granulocytic leukemia and Ph + ALL in the US in December 2012 and in the EU in July 2013 [28]. **1e** has been shown to be a potent inhibitor of Bcr-Abl. It has demonstrated clinical activity in both Bcr-Abl wild-type and mutant CML, including anti-T315I mutation activity [21]. Here, we briefly describe the development history of **1e**, where researchers targeted the inactive DFG domain of ABL proteins based on the 9-[anyenyl]purine core (Figure 1). Compound **1a** had a weak inhibitory effect on ABL T315I with an IC_50_ of 14,142 nM. To further reduce the steric clash with the main backbone of the isoleucine side chain, the double bond linker of **1a** was replaced with a triple bond to obtain compound **1b** (T315I kinase IC_50_ = 524 nM). Compared to **1a**, **1b** showed a significant increase in kinase activity and anti-proliferative activity (tested in Ba/F3 cells). However, **1b** showed poor pharmacokinetics (PK) performance in rats. To further improve the bioavailability of compounds, the researchers further modified the structure of **1b** to obtain compound **1c** (T315I kinase IC_50_ = 102 nM). The experimental results showed that the PK properties of **1c** were considerably improved with an oral bioavailability (*F*) of 42.4% at dose of 15 mg/kg in rats, but the anti-proliferative activity was low against ABL T315I Ba/F3 cells (IC_50_ = 471 nM). To further enhance the anti-proliferative potency, compound **1d** (T315I kinase IC_50_ = 56 nM) was obtained by introducing *N*-methylpiperazine at C-4 on the 3-trifluoromethylbenzene ring. **1d** showed a 2-fold increase in anti-T315I mutant kinase activity and an approximately 20-fold increase in potency (IC_50_ = 26 nM) in inhibiting the growth of Ba/F3 cells of Bcr-Abl T315I, and **1d** also exhibited desirable PK properties (*F* = 29.0%). Encouraged by the overall performance of **1d**, structure–activity relationships (SARs) were further explored around **1d** by introducing an additional N atom to the pyridine ring to reduce the lipophilicity of **1d** (cLogP = 6.69), targeting the final compound **1e** (cLogP ≈ 4.69, T315I kinase IC_50_ = 40 nM; T315I Ba/F3 IC_50_ = 1.2 nM). Compared to **1d**, **1e** not only showed better enzymatic activity levels and anti-cell proliferation activity but also showed more desirable results in PK properties [29]. The X-ray structure of the Ponatinib-Bcr-Abl complex shows that **1e** can occupy the ATP-binding pocket properly, where N-3 in imidazo [1,2-*b*]pyridazine can form hydrogen bond interactions with residue MET318 at a distance of 1.9 Å. The -NH and -C=O of the amide group can form hydrogen bonds with the carbonyl oxygen atom of GLU286 and the -NH of ASP381 at distances of 2.6 and 1.8 Å, respectively. The N atom of the *N*-methylpiperazine portion can form hydrogen bonds with residues HIS316 and ILE360 at distances of 3.4 and 2.9 Å, respectively, and also form salt bridges with ASP381.

Taken together, **1e** is a potent Bcr-Abl inhibitor that has been approved for the treatment of CML patients who are resistant or intolerant to Imatinib, Dasatinib, or Nilotinib, and studies have confirmed that **1e** can be effective in a variety of other cancers [30]. In the future, the clinical safety and patient tolerability profile of **1e** need to be further explored.

### 2.2. Bruton’s Tyrosine Kinase (BTK) Inhibitors

Bruton’s tyrosine kinase (BTK), a member of non-receptor tyrosine kinase belonging to the Tec family kinases [31], is a vital component in the B-cell receptor signaling pathway. BTK is widely expressed in different types of malignant hematological diseases and is involved in the proliferation, differentiation, and apoptosis of B cells [32]. BTK has emerged as a promising target for the treatment of multiple diseases, especially B-cell-related malignancies, and the first-, second-, and third-generation BTK inhibitors have been developed over the past decades [33,34]. Small-molecule BTK inhibitors include both covalent and non-covalent inhibitors, of which the first- and second generations are predominantly covalent, while the third generation is mostly non-covalent.

**Figure 2:**
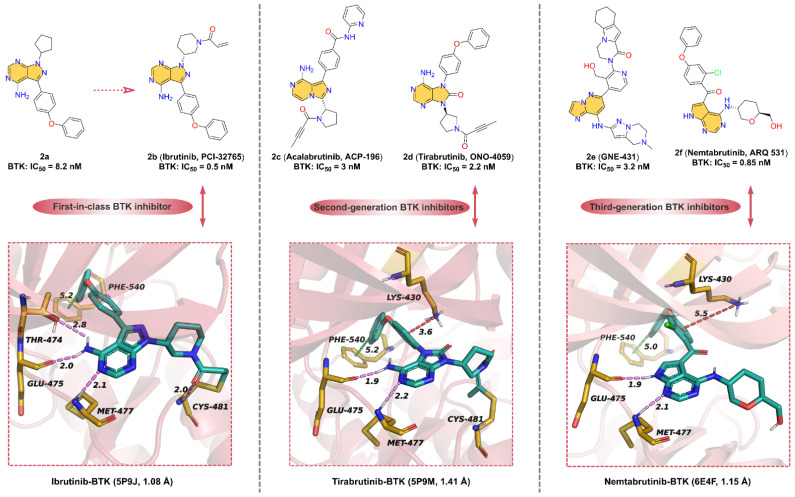
Representative azaindole/oxindole core-containing BTK inhibitor in clinical applications (**2e, GNE-431**, has not yet entered into clinical studies).

Here, we focus on small-molecule BTK inhibitors with azaindole or oxindole core in clinical applications (Figure 2). **2b** (**Ibrutinib**, **PCI-32765**), the first-in-class BTK inhibitor, is an orally available, highly potent, and irreversible ATP-competitive kinase inhibitor for the treatment of mantle cell lymphoma (MCL) and chronic lymphocytic leukemia (CLL) [35,36]. The development of **2b** was first reported by Pan. et al. [37], who identified compound **2a** had moderate inhibitory activity against BTK with an IC_50_ of 8.2 nM but was poorly selective for BTK. To further give compounds with potent kinase activity and better selectivity for BTK, a diverse array of Michael acceptor groups was introduced into **2a**. Encouragingly, **2b** exhibits the strongest inhibition of BTK kinase with an IC_50_ of 0.5 nM and a significant improvement in selectivity for BTK. **2b**, which contains an acrylamide motif, can form a covalent bond with the residue CYS481 to enhance BTK selectively. In addition to the formation of a covalent bond, **2b** can also form non-covalent bond interactions with key residues around the ATP-binding pocket of BTK. As shown in Figure 2, the cocrystal structure of **2b** with BTK [38] reveals that the 4-amino-1*H*-pyrazolo [3,4-*d*]pyrimidin scaffold can form tridentate hydrogen bonds with the hinge residues THR474, GLU475 and MET477, with distances of 2.8, 2.0, and 2.1 Å, respectively. In addition, the phenyl of **2b** occupies the hydrophobic pocket in the N-lobe of BTK and displays edge-to-face pi–pi interactions with residue PHE540. Overall, the azaindole core of **2b** is able to form hinge hydrogen bonds with key residues in the hinge region, stabilizing it at the ATP-binding site.

Subsequent studies have found that oral **2b** has serious adverse effects, limiting its application in cancer therapeutics [39,40,41]. To overcome the drug resistance and off-target side effects of **2b**, selective second-generation BTK inhibitors have been developed. **2c** (**Acalabrutinib**, **ACP-196**), a second generation BTK inhibitor, has better selectivity and safety than the first-in-class BTK inhibitor **2b** and improved off-target effects [42]. **2c** is structurally related to **2b**, used in the treatment of a variety of hematologic malignancies and solid tumors, as well as potentially in the treatment of rheumatoid arthritis (RA) [43]. The key structural components of **2c** include the 8-amino-imidazo [1,5-*a*]pyrazin scaffold involved in the formation of hinge hydrogen bonds, the 2-pyridylbenzoamide moiety forming hydrophobic interactions at the binding site, and 2-butylamide part covalently binding to Cys481. Unlike the acrylamide functional group in the previously reported inhibitors, the covalent motif of **2c** is 2-butylamide. It is speculated that due to this unique active 2-butylamide moiety, **2c** exhibits lower reactivity than acrylamides **2b**, which may help to minimize the inhibition of off-target cysteine kinases [42]. Compared with **2b**, **2c** only inhibited BTK, BMX, and ERBB4 at clinically relevant concentrations, which explains the higher specificity of the BTK inhibitor [44]. 

**2d** (**Tirabrutinib**, **ONO-4059**), an analog of compound **2b** scaffold, is a potent, second-generation inhibitor of BTK (IC_50_ = 2.2 nM) [45]. **2d** can inhibit BTK activity by blocking the autophosphorylation of residue TYR223 [33]. Although **2d** and **2b** display similar binding models, **2d** is significantly more selective and effective. As depicted in Figure 2, the parent core of the molecular structure of **2d** is a 6-5-membered, fused, heterocyclic ring (oxindole moiety) that can form hydrogen bonds with hinge residues Glu475 and Met477 with distances of 1.9 and 2.2 Å, respectively. Furthermore, another model for the interaction between **2d** and BTK is the covalent interaction between 2-butylamide and the residue CYS481. The phenoxyphenyl extends into the hydrophobic pocket and forms pi–pi interactions with residue PHE540, such as **2b**.

**2e** (**GNE-431**) is a potent, selective, and noncovalent BTK inhibitor with IC_50_ of 3.2 nM and 2.5 nM for wild-type BTK and C481S mutants, respectively [46,47]. **2e**, unlike covalent inhibitors such as **2b**, does not form a covalent bond with CYS481 and potently inhibits **2b**-resistant BTK C481S mutant in vitro and in vivo. The computational binding model of BTK to **2e** suggests that the imidazo [1,2-*b*]pyridazin-8-amine moiety can form hinge hydrogen bonds with the hinge residues and that the tetrahydropyrazolo [1,5-*a*]pyrazine group has a distinctly different binding orientation than other BTK inhibitors [46]. Due to the unique binding pose of **2e**, this allows it to inhibit wild-type BTK and the mutants, such as C481S, C481R, T474I, and T474M (IC_50_ = 7.5–10 nM). These mutant residues have no spatial overlap with **2e** at the binding site. 

In response to emerging drug resistance of **2b**, a third-generation orally bioavailable reversible BTK inhibitor, **2f** (**Nemtabrutinib**, **ARQ 531**), has been identified [48]. **2f** shows potent inhibition of both wild-type and C481S-mutant BTK with IC_50_ of 0.85 and 0.39 nM, respectively. Additionally, **2f** has significant anti-proliferative activity in vitro against many hematological malignant cell lines, including Ibrutinib-resistant cell lines [49,50]. As shown in Figure 2, the crystal structure of BTK with **2f** [49] at 1.15 Å resolution is similar to that of BTK with **2b**. The pyrrolo [2,3-*d*]pyrimidine core can form hinge hydrogen bonds with key residues GLU475 and MET477 in the hinge region. Similar to **2b**, phenoxyphenyl group of **2f** occupies the hydrophobic pocket of the ATP-binding site, forming pi–pi stacking interactions and pi–cation interactions, respectively. It is worth mentioning that **2f** does not form a covalent bond with CYS481; the polar tetrahydropyran methanol motif is exposed in the solvent region, indicating that the C481S-mutat BTK does not affect the binding of **2f**. 

### 2.3. Cyclin-Dependent Kinases 4/6 (CDK4/6) Inhibitors

**Figure 3:**
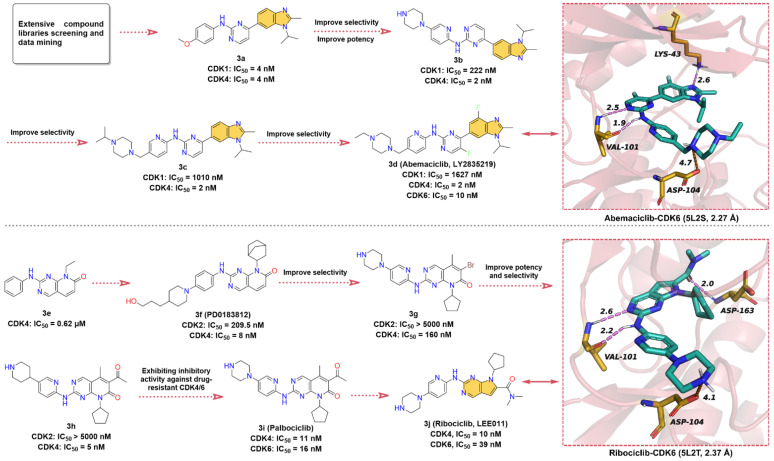
Key medicinal chemistry optimization of **Abemaciclib** and **Ribociclib** along with the binding model with CDK6.

Cyclin-dependent kinases 4/6 (CDK4/6) are members of the serine/threonine kinases family that play pivotal roles in the biological processes that regulate the cell cycle. Aberrant activation or dysregulation of CDK 4/6 is closely associated with cancer development and progression [51]. First-generation pan-CDK inhibitors and second-generation multi-CDK inhibitors are poorly selective and have highly toxic side effects [52]. As a result, a new generation of selective CDK4/6 inhibitors (including **Abemaciclib**, **Palbociclib**, and **Ribociclib**, as shown in Figure 3) have been developed, aiming to improve the selectivity and reduce the adverse side effects of the drugs [53].

The hit compound 2-anilino-2,4-pyrimidinyl-benzimidazole scaffold (**3a**), which showed strong inhibitory activity against both CDK1 and CDK4 with IC_50_ value of 4 nM, was obtained through screening and data mining of extensive chemical libraries [54]. Subsequently, **3a** was subjected to SARs studies to improve its inhibitory potency and selectivity against CDK4. This process mainly involves the replacement of the phenyl ring in aniline with pyridine and the introduction of the piperazine ring to obtain **3b**, which improves the selectivity for CDK4 and its IC_50_ for CDK1 inhibition increases from 4 nM to 222 nM. Modification of the piperazine fragment of **3b** gives **3c** (CDK4 IC_50_ = 2 nM, CDK1 IC_50_ = 1010 nM), whose activity against CDK4 is maintained while selectivity is further improved. To enhance the specificity of **3c** for CDK4/6, the introduction of fluorine atoms on pyrimidines and benzimidazoles resulted in the approved drug **3d** (**Abemaciclib**, **LY2835219**). **3d** has a greatly reduced inhibitory activity against CDK1 (IC_50_ = 1627 nM) and shows potent inhibitory efficacy on CDK4/6 with IC_50_ values of 2 and 10 nM, respectively [55]. Crystal structure analysis of **3d** with CDK6 (Figure 3, upper right) yields that the 2-amino-pyrimidine fragment forms bidentate hydrogen bonds with the hinge residue VAL101 (1.9 & 2.5 Å), and the benzimidazole moiety binds to the hydrophobic region of the ATP-binding site and forms a hydrogen bond interaction with LYS43 at a distance of 2.6 Å [56]. In addition, the piperazine moiety of **3d** is exposed to the solvent region, forming a salt bridge interaction with ASP104. These diverse non-covalent bond interactions enable **3d** to bind competitively to the ATP-binding site of CDK6.

Pyrido [2,3-*d*]pyrimidin-7-one analogs are reported to have good inhibitory activity against CDKs, among which, 2-phenylamino-pyrido [2,3-*d*]pyrimidin-7-one (**3e**) shows moderate inhibitory activity against CDK4 with an IC_50_ of 0.62 μM [57]. Chemical modification of **3e** resulted in the identification of **3f** (**PD0183812**) [58], which shows potent inhibition and high selectivity for both CDK4 (IC_50_ = 8 nM) and CDK6 (IC_50_ = 13 nM), but poor selectivity over other CDK isoforms (CDK2, IC_50_ = 209.5 nM). SARs studies have shown that the selectivity of the 2-aminopyridine fragment on the quinazoline core is higher than that of the aniline moiety. Thus, the modification of the aniline component of **3f** leads to **3g** with higher selectivity against CDK4/6. Intriguingly, replacing the -Br on the quinazoline scaffold with an acetyl group gives **3h** with a 32-fold increase in its inhibitory activity against CDK4. Replacement of the piperidine moiety of **3h** with piperazine affords **3i** (**Palbociclib)** [59], a highly specific CDK4/6 inhibitor with IC_50_ values of 11 and 16 nM, respectively. In 2010, Novartis, in collaboration with Astex, reported **3j** (**Ribociclib**, **LEE011**), an orally active, highly specific CDK4/6 inhibitor with IC_50_ values of 10 and 39 nM, respectively [60,61]. To the best of our knowledge, the development course of **3j** has not been disclosed [55,62]. Given the structural similarity and the chronological order of launch, we assume that **3j** was designed with **3i** as the lead compound. In short, the 6,6-membered fused quinazoline ring of **3i** is reduced to a 6,5-membered fused pyrrolo [2,3-*d*]pyrimidine scaffold. The X-ray cocrystal structure of human CDK6 and **3j** (Figure 3, lower right) illustrates that the pyrrolo [2,3-*d*]pyrimidine group can form a hinge hydrogen bond with VAL101, and the -NH of the pyrimidine amino group also forms a hydrogen bond with hinge residue VAL101. Same as with **3i**, the two side chains of **3j** can form salt bridge interaction and hydrogen bond interaction with ATP-binding site residues ASP104 and ASP163, respectively.

### 2.4. Colony-Stimulating Factor 1 Receptor (CSF1R) Inhibitors

Receptor tyrosine kinases (RTKs) play an imperative role in maintaining homeostasis in vivo. RTKs provide important pathways for cellular communication, maintain signal transduction for normal cellular processes, and provide surface receptors for many hormones and growth factors [63]. The human genome contains 58 RTKs, which are divided into different groups according to the homology of their active sites and the similarity of ligands [64]. Mutations in receptor structure and ligand overexpression facilitate the emergence and development of several types of cancers. Colony-stimulating factor 1 receptor (CSF1R), also known as c-FMS, CD115, or M-CSFR, is one of the most important receptors in type III RTKs and is increasingly becoming a promising target for cancer therapy [65]. Activation of CSF1R promotes survival, proliferation, and differentiation of macrophage and monocyte lineages. Monocytes in the tumor microenvironment can differentiate into tumor-associated macrophages (TAMs) that allow cancer cell growth, metastasis, angiogenesis, and especially local immunosuppression. Tumor-released colony-stimulating factor 1 (CSF1) binds to CSF1R and promotes macrophage proliferation, survival, and differentiation. Overexpression of CSF1 and CSF1R in a variety of tumors, including breast cancer, is associated with poor patient prognosis, thus suggesting that overexpression of CSF1R is associated with diseases such as cancer [66,67,68,69]. Therefore, the development of kinase inhibitors targeting CSF1R could be an effective cancer treatment strategy [70].

**Figure 4:**
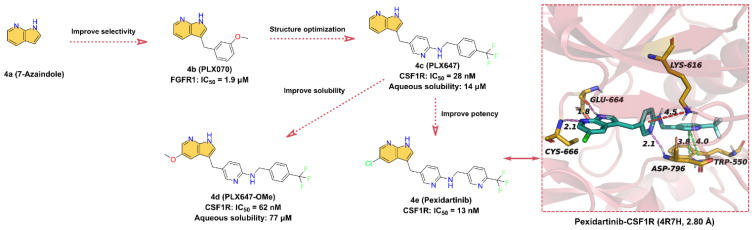
Key medicinal chemistry optimization of **Pexidartinib** along with the binding model with CSF1R.

**4e** (**Pexidartinib**, Figure 4) is an oral antitumor small-molecule drug that potently inhibits CSF1R with an IC_50_ of 13 nM for the treatment of tenosynovial giant-cell tumors [71,72]. **4a** (**7-azaindole**) is identified as a unique kinase inhibitor scaffold by screening over 20,000 low-molecular-weight compounds against multiple kinases [73,74]. **4b** (**PLX070**) is derived by introducing a 3-methoxybenzyl group at the 3-position of **4a**. The crystal structure shows that **4b** is able to bind at the ATP-binding site of FGFR1 (fibroblast growth factor receptor 1, IC_50_ = 1.9 μM) and that the 7-azaindole moiety can form two hydrogen bonds with the hinge residues, indicating that **4b** is an effective ATP-competitive kinase inhibitor designed building block [74]. The researchers maintained the 3-methylene linker of **4b** and employed pyridine in place of the benzene ring to provide the endocyclic nitrogen as a hydrogen bond acceptor to replace the methoxy of the exocyclic ring and introduced trifluoromethyl-benzylamine to obtain **4c** (**PLX647**) (IC_50_ = 28 nM for CSF1R) [75]. To further optimize the structure, **4d** (**PLX647-OMe**) is obtained by introducing a methoxy group at the 5-position of the 7-azaindole of **4c**. The inhibitory efficacy of **4d** against SCF1R was slightly reduced (IC_50_ = 62 nM), but the aqueous solubility is significantly enhanced, from 14 μM for **4c** to 77 μM [75]. To further optimize the structure of **4d** to enhance the inhibitory potency of SCF1R, **4e** (**Pexidartinib**) is obtained by replacing the -OCH_3_ group with -Cl, and the inhibition of CSF1R kinase is improved with an IC_50_ of 13 nM [70]. As shown in Figure 4, **4e** binds into the ATP-binding pocket of CSF1R and makes direct contact with surrounding key residues [72]. Specifically, the -NH and -N on the 7-azaindole scaffold form two hydrogen bonds with hinge residues GLU664 and CYS666 at distances of 1.8 and 2.1 Å, respectively. In addition to the pi–cation interaction between the middle pyridine group and residue LYS616, the polar nitrogen of the pyridine is also involved in the formation of a hydrogen bond with residue ASP796 at a distance of 2.1 Å. Additionally, the 3-fluoromethylpyridine block in the tail can interact with the indole group of the residues TRP550 to form face-to-face pi–pi interactions.

### 2.5. Human Epidermal Growth Factor Receptor (HER) Inhibitors

**Figure 5:**
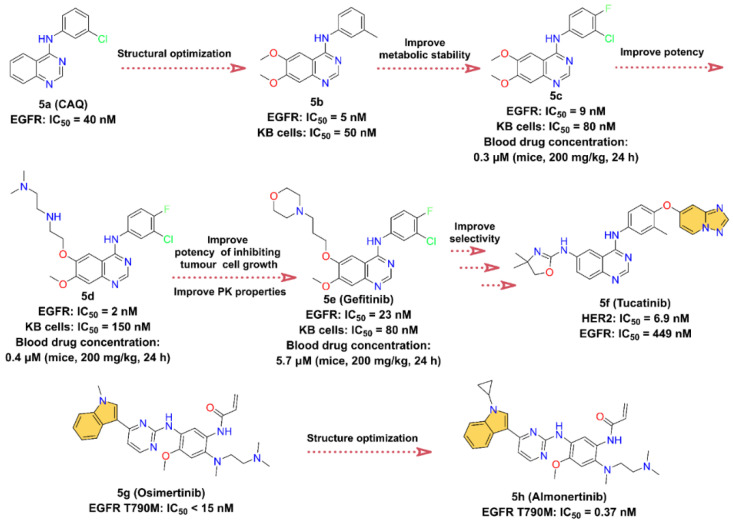
Key medicinal chemistry optimization of **Tucatinib**, **Osimertinib**, and **Almonertinib**.

Functional activation of the human epidermal growth factor receptor 2 (HER2) gene promotes the development of cancer. The main mechanism of HER2 activation in human breast and gastric cancers is HER2 gene amplification, leading to overexpression of its protein on the cell membrane, which is associated with disease recurrence and short overall patient survival [76,77]. Therefore, the development of specific HER2 antagonists is of great therapeutic importance in oncology [76]. Due to the high expression of HER2 in tumors such as breast cancer, several novel therapeutic strategies, including the administration of small-molecule inhibitors and monoclonal antibodies, have significantly improved patient survival. Currently, the main small-molecule drugs that effectively target HER2 are ATP-competitive inhibitors, including **Tucatinib**, Lapatinib, Neratinib (HKI-272), and Pyrotinib, which compete with ATP to block phosphorylation and activate downstream signaling cascades [78]. Notably, **Tucatinib** (**5f**) is a selective HER2 inhibitor containing azaindole scaffold, which is also the focus of the next discussion (Figure 5).

In the course of investigating the catalytic mechanism of epidermal growth factor receptor (EGFR) tyrosine kinase using a structure-based searching approach, a potent ATP-competitive inhibitor of EGFR, 4-(3-chloroanilino)quinazoline **5a** (**CAQ**), with a Ki of 16 nM and an IC_50_ of 40 nM, was identified [79,80]. **5a** is the first representative of a new structural class of anilinoquinazoline tyrosine kinase inhibitors [79]. We speculate that the optimization of the structure of **5a** yields **5b** with stronger enzyme inhibitory activity (IC_50_ = 5 nM) and cell proliferation inhibitory activity (KB cells, IC_50_ = 50 nM). However, the phenylmethyl of **5b** is easily oxidized by metabolism in vivo. To improve the metabolic stability of **5b**, an F atom is introduced at the 4-position of the phenyl group, and the methyl is replaced with a Cl atom to close the metabolic site to obtain **5c**. **5c** inhibits EGFR with an IC_50_ value of 9 nM, with a slight loss of inhibitory potency but improved PK properties, such as its half-life (t_1/2_) is increased by approximately three times [81]. Optimization of the 6-position of the quinazoline ring at **5c** gives **5d**, which shows a stronger inhibitory effect for EGFR with an IC_50_ of 2 nM, but the proliferation inhibitory activity against KB cells is reduced (IC_50_ = 150 nM). In addition, there is a slight improvement in the PK properties at **5d**, with blood drug concentrations in mice at 24 h elevated from 0.3 μM at **5c** to 0.4 μM at a dose of 200 mg/kg. To further enhance the cell proliferation inhibitory activity as well as the bioavailability of **5d**, the morpholine group is introduced to obtain **5e** (**Gefitinib**). The blood concentration of **5e** is further increased at 5.7 μM after 24 h of administration, while the IC_50_ of **5e** inhibition on KB cells is 80 nM, which is also enhanced. **5e** has good oral bioavailability and is a first-line treatment for patients with metastatic non-small-cell lung cancer (NSCLC) [81,82,83]. **5f** (**Tucatinib**) is initially identified through a small-molecule discovery effort on HER2 and EGFR inhibitors. It was discovered by Array BioPharma Inc. and received FDA approval in 2020 to treat advanced unresectable or metastatic HER2-positive breast cancer [84]. **5f** is a highly selective HER2 inhibitor containing a quinazoline core such as **5e**, with IC_50_ values of 6.9 nM for HER2 and 449 nM for EGFR. Therefore, it is speculated that **5f** is optimized from **5e** [85,86].

EGFR is also known as HER1, and its overexpression plays an important role in a variety of cancers, such as squamous-cell carcinoma of the lung, glioblastoma, and epithelial tumors of the head and neck [87]. **5g** (**Osimertinib**) is a third-generation EGFR inhibitor developed by AstraZeneca to overcome EGFR T790M mutation-related resistance [88]. It is highly selective for EGFR-activating mutations and EGFR T790M mutation [89]. In November 2015, it was approved by the FDA to treat metastatic EGFR T790M mutation-positive NSCLC, with IC_50_ less than 15 nM for EGFR T790M [88,90,91]. Innovative optimization on the structure of **5g**, **5h** (**Almonertinib**) is obtained by substituting the -CH_3_ on the N atom of the indole group and introducing cyclopropyl [92,93]. Surprisingly, the problem of high toxicity and poor selectivity of **5g**’s metabolites is solved [92,94]. In March 2020, **5h** was approved by the National Medical Products Administration (NMPA) in China for the treatment of advanced EGFR T790M + NSCLC [93]. It is the third-generation EGFR inhibitor developed by Jiangsu Hansoh Pharmaceutical Co. [93], with IC_50_ values of 0.37 ± 0.04 nM, 0.21 ± 0.10 nM, 0.29 ± 0.10 nM, and 3.39 ± 0.53 nM for T790M, Del19/T790M, L858R/T790M, and EGFR WT, respectively [92].

### 2.6. Janus Kinases (JAK) Inhibitors

Janus kinases (JAK) belong to the family of non-receptor kinase tyrosine kinases, which consists of four members together, including JAK1, JAK2, JAK3, and TYK2 [95]. JAK1, JAK2, and TYK2 are commonly expressed, while JAK3 is mainly in hematopoietic cells [96]. In the 1990s, JAKs were found to have a role in cytokine signaling pathways [97]. JAKs can transmit signals from cell membrane receptors to the signal transducer and activator of the transcription (STAT) family, the JAK/STAT signaling pathway, and are closely associated with cancer [98] and inflammatory diseases [99]. In 2005, researchers identified in patients with myeloproliferative neoplasms V617F mutations in JAK2, including myelofibrosis, true erythroblastosis, and primary thrombocytopenia [100]. Studies have shown that excessive activation of the JAK/STAT pathway has been detected in several solid cancers (lung, breast, head, and neck) and hematologic malignancies (multiple myeloma, lymphoma, and non-lymphoma), as well as in acute leukemia [101]. Increasing evidence suggests that inhibition of JAK expression can treat inflammation or cancer that is dependent on JAK/STAT pathway activation, and such, this has attracted an increasing number of scholars and pharmaceutical companies to invest in the development of small-molecule inhibitors targeting JAK [102].

**Figure 6:**
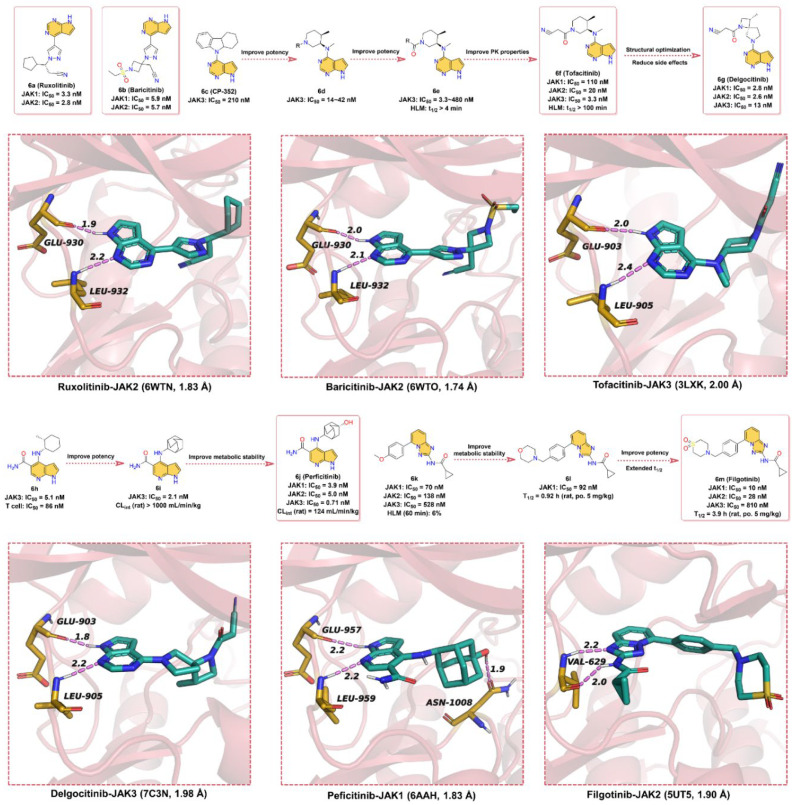
Key medicinal chemistry optimization of **JAKs** inhibitors along with the binding models with **JAKs**.

Herein, we describe small-molecule JAK inhibitors containing azaindole core in clinical applications (Figure 6). To date, there are six JAK inhibitors with azaindoles structural motifs have been applied in the clinic, namely **Ruxolitinib**, **Baricitinib**, **Tofacitinib**, **Delgocitinib**, **Peficitinib,** and **Filgotinib**. Among them, **6a** (**Ruxolitinib**), the first FDA-approved JAK1/2 inhibitor, kills tumor cells by enhancing apoptosis and inducing autophagy [103]. However, some side effects, such as anemia, thrombocytopenia, and neutropenia, may occur as **6a** also inhibits JAK2 [104]. As shown in Figure 6, **6a** has a pyrrolo [2,3-*d*]pyrimidin core, in which the -NH of the pyrrole can form a hinge hydrogen bond with residue GLU930 at a distance of 1.9 Å, while the N atom on the pyrimidine group can form a hydrogen bond with hinge residue LEU932 at a distance of 2.2 Å. Overall, the azaindole fragment of **6a** is able to form hinge hydrogen bonds with key residues in the ATP-binding site of JAK2, thereby competing with ATP for the binding pocket.

**6b** (**Baricitinib**) is a JAK1 and JAK2 inhibitor developed by Eli Lilly and Company for the treatment of RA [105], atopic dermatitis, and systemic lupus erythematosus. **6b** has an IC_50_ of 5.9 nM and 5.7nM for in vitro kinase activity testing on JAK1 and JAK2, respectively, and does not inhibit c-Met or CHK2. Attempts have been made to approve **6b** as a therapeutic agent for RA in Japan and the United States, but the US FDA is currently unable to approve the drug because further clinical data are needed to determine the appropriate dose and to better characterize the safety of the compound [106]. As shown in Figure 6, the chemical parent core of **6b** is also the pyrrolo [2,3-*d*]pyrimidin scaffold, and the binding pattern between **6b** and JAK2 is similar to that of **6a**; that is, the parent scaffold of **6b** forms bidentate hydrogen bond interactions with key residues GLU930 and LEU932 in the hinge region of JAK2.

In 1996, Pfizer screened its compound library for small-molecule inhibitors targeting JAK3 and identified the pyrrolo [2,3-*d*]pyrimidin-type derivative **6c** (**CP-352**), which has a JAK3 kinase inhibition of 210 nM and a short half-life in human liver microsomes [107]. To further enhance the potency and extend the half-life in human liver microsomes, a variety of substituents, including cyclic, lipophilic, and amino groups, are used to explore compounds with better JAK3 inhibitory activity. Surprisingly, *N*-methylcycloalkyl analogs (**6d**) showed better enzymatic activity against JAK1 and some improvement in T-cell activity, suggesting that simultaneous inhibition of JAK1 and JAK3 may be the key to improving cellular potency. The amide group with better hydrophilicity was introduced to **6d** as a linker to obtain **6e** (human liver microsome, HLM, t_1/2_ > 4min). To further improve the PK properties, a small polar cyano group was introduced next to the amide group to finally give **6f** (**Tofacitinib**, HLM, t_1/2_ > 100 min), which can maintain a favorable balance in terms of JAK kinases selectivity and druggability [107]. The IC_50_ values of **6f** for JAK3, JAK2, and JAK1 kinases inhibitory effect are 3.3, 20, and 110 nM, respectively, and **6f** citrate has anti-infective activity [108]. **6f**, a pan-JAK inhibitor developed by Pfizer, was approved by the US FDA in 2012 for the clinical treatment of moderate-to-severe RA that cannot be treated with methotrexate [16]. As depicted in Figure 6, the pyrrolo [2,3-*d*]pyrimidin scaffold of **6f** occupies the ATP-binding pocket of JAK3 and forms two hydrogen bonds with hinge residues GLU903 and LEU905 at distances of 2.0 and 2.4 Å, respectively.

In the clinical phase II trial, **6f** showed adverse effects such as headache and nausea [109]. The researchers developed **6g** (**Delgocitinib**) based on the structure of **6f** to reduce these side effects. **6g** is a potent, orally available, pan-JAK inhibitor with IC_50_ values of 2.8, 2.6, 13, and 58 nM for JAK1, JAK2, JAK3, and Tyk2, respectively, which was approved for the treatment of atopic dermatitis in Japan [110]. As depicted by the X-ray cocrystal structure of JAK with **6g**, the -NH of pyrrole in the pyrrolo [2,3-*d*]pyrimidin core of **6g** can form a hinge hydrogen bond with residue GLU903 at a distance of 1.8 Å, and the N atom of pyrimidine serves as hydrogen bond acceptor to form hydrogen bond interactions with hinge residue LEU905 at a distance 2.2 Å.

In 2007, the patent reported that **6j** (**Peficitinib**) is a particularly potent JAK inhibitor against JAK3 with an IC_50_ of 0.71 nM, while the inhibitory activity against JAK1, 2, and TYK2 kinases is 3.9, 5.0, and 4.8 nM, respectively [111]. **6j** has reached late-stage clinical trials for the treatment of RA [112]. **6j** was optimized from the lead compound **6h**, which exhibited potent JAK3 inhibitory activity (IC_50_ = 5.1 nM) and moderate T-cell proliferation inhibitory activity (IC_50_ = 86 nM). However, **6h** showed poor metabolic stability in liver microsomes and in vivo PK profiles. To further improve the inhibitory potency of JAK3, **6i** was obtained by replacing cyclohexane with adamantane moiety. **6i** showed stronger inhibition of JAK3 with an IC_50_ of 2.1 nM, but its PK properties were poor, and its plasma clearance (CL) was greater than 1000 mL/min/kg in rats. Finally, the polar group -OH was introduced in the C4-adamantyl of **6i** to obtain **6j** to reduce lipophilicity and thus improve metabolic stability (CL = 124 mL/min/kg). According to the binding model between **6j** and JAK1, the -NH and N-atom on the pyrrolo [2,3-*d*]pyridine substructure can form bidentate hinge hydrogen bonds with residues GLU957 and LEU959, both at a distance of 2.2 Å. Unlike other JAK inhibitors, the -OH on adamantane can form a strong hydrogen bond interaction with residue ASN1008 with a distance of 1.9 Å. It is speculated that this may account for the particularly good activity of **6j** against JAK3. 

In 2013, researchers obtained hit compound **6k** against the JAK family by high-throughput screening, but with low to medium inhibitory activity (JAK1 IC_50_ = 70 ± 14 nM, JAK2 IC_50_ = 138 ± 22 nM, JAK3 IC_50_ = 528 ± 82 nM, TYK2 IC_50_ = 519 ± 55 nM) [113]. Moreover, **6k** shows low metabolic stability; only 6% of the prototype drug remains in human liver microsomes at 60 min. In the modification of **6k**, the researchers found that the potency of enzyme activity was increased when the substituent on the phenyl ring was large enough to contact the glycine-rich ring. When the methoxy on the benzene ring is replaced with morpholine (**6l**), it is found that the t_1/2_ in human microsomes could be prolonged, but the inhibitory effect on JAK1 remains low (IC_50_ = 92 ± 12 nM). Encouragingly, the replacement of the morpholine with cyclic sulfone fragment resulted in **6m** (**Filgotinib**), which has greatly improved potency against JAK1 (IC_50_ = 10 ± 0.8 nM) and also possessed more desirable PK properties (t_1/2_ = 3.9 h) [114]. Based on the X-ray cocrystal structure, **6m** binds into the ATP-binding site, the N-atom in triazolo [1,5-*a*]pyridine scaffold, and the -NH in the neighboring amide form bidentate hinge hydrogen bond interactions with the residue VAL629 at distances of 2.2 and 2.0 Å, respectively. The benzene ring forms hydrophobic interactions, while the partial fragment of thiomorpholine dioxide extends to the solvent-exposed region. The favorable PK profile, especially the oral availability in different animal species, as well as the activity in preclinical models, led to the development of this inhibitor in clinical application [115,116].

Briefly, all six of these JAK inhibitors are ATP-competitive inhibitors that bind to the ATP-binding pockets. Current clinical JAK small-molecule inhibitors have progressed to higher levels of potency against JAK, and these pan-JAK inhibitors have been found to cause side effects in patients with different conditions in the clinic. Further improvements in the selectivity of JAK kinases are essential to reduce side effects, while attention should also be paid to the PK of the drug in vivo, which will be the focus of the development of the next generation of JAK kinase inhibitors.

### 2.7. BRAF Inhibitors

BRAF is a member of the serine/threonine kinase RAF family (ARAF, BRAF, and CRAF) and is an important part of the RAS/RAF/MEK/ERK mitogen-activated protein kinase (MAPK) signaling pathway [117,118]. Among these, the MAPK cascade is a key signaling pathway involved in the regulation of normal cell proliferation, survival, and differentiation [119], and oncogenic mutations of this pathway are commonly observed in numerous cancers [120]. Since RAS mutations are found in approximately 30% of cancers, RAS is considered a central therapeutic target of this pathway, while the physiological signaling of RAS is mainly caused by BRAF [121]. Studies have demonstrated that regulation of the MAPK signaling pathway by targeting BRAF kinase is already the standard of care for patients with metastatic melanoma containing BRAF mutations [122,123]. Since the identification of BRAF-V600E as a drug target, many researchers have been attracted to developing specific kinase inhibitors against RAF.

**Figure 7:**
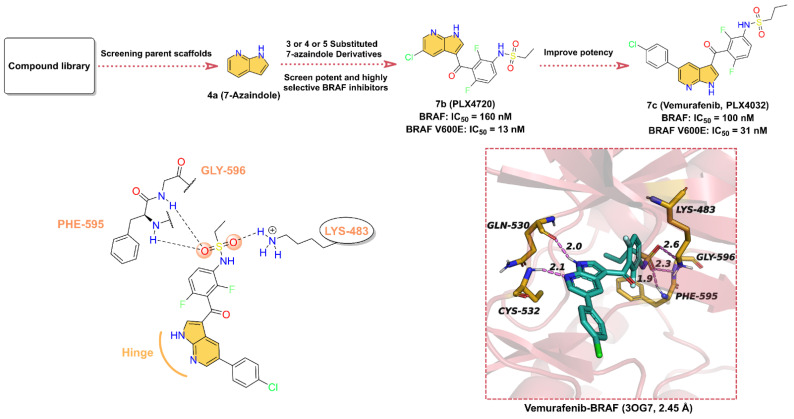
Key medicinal chemistry optimization of **BRAF** inhibitors along with the binding models with **BRAF**.

Currently, the inhibitors targeting RAF have been developed into the third generation [124]. In this section, our perspective is focused on **7c** (**Vemurafenib**, **PLX4032**), an ATP-competitive RAF inhibitor containing azaindole scaffold, which was approved by the US FDA in 2011 for the treatment of patients with BRAF-V600E metastatic melanoma (Figure 7). **7c** has high activity against BRAF-V600E with an IC_50_ of 31 nM and effectively inhibits ERK phosphorylation in tumor cell lines bearing BRAF-V600E [125]. The discovery of **7c** was based on a 7-azaindole(**4a**) scaffold obtained from kinases screening experiments of a library of more than 20,000 compounds [74]. Subsequently, a library of mono- and disubstituted derivatives was constructed using **4a** as the parent scaffold, and screening of this compound library yielded that **7b** (**PLX4720**) exhibited excellent potency against BRAF-V600E with an IC_50_ of 13 nM and high selectivity for other kinases. The IC_50_ for the in vitro proliferation inhibitory activity of **7b** against melanoma A375 (BRAF-V600E) cells was 500 nM. We presume that in order to further obtain drugs with better anticancer activity, the researchers used a rational structure-based drug design approach to introduce p-chlorophenyl moiety at the 5-position of the azaindole scaffold to obtain **7c**, which showed significantly enhanced anticancer effects on A375 cells (IC_50_ = 310 nM) [126]. As shown in Figure 7, **7c** is able to well occupy the ATP-binding pocket of BRAF, where the hydrogen bonds of azaindoles to the hinge residues anchor the structure [127]. Specifically, N1 serves as a hydrogen bond donor to form a hydrogen bond with the backbone carbonyl of GLN530, while N7 provides a hydrogen bond acceptor to produce a hydrogen bond with the backbone amide of CYS532 at distances of 2.0 and 2.1 Å, respectively. 3-chlorobenzene extends toward the activation segment; the *N*-(2,4-difluorophenyl)propanesulfonamide fragment, on the other hand, is binding in the hydrophobic region, where the two O atoms of the sulfanedione group can form three hydrogen bonds with residues LYS483, PHE595, and GLY596 at distances of 2.6, 1.9, and 2.3 Å, respectively.

In conclusion, **7c** is a very effective drug for the treatment of unresectable metastatic melanoma, with clinical manifestations of tumor regression in 85% of patients, but half of the patients also experienced resistance problems [128]. It has been reported that increased RAF dimerization may be responsible for the clinical resistance to RAF inhibitors [129,130,131]. Since compensatory survival signals occur upon BRAF inhibition, it has been shown that resistance in these cell lines can be overcome in vitro when MEK inhibitors are used in combination [132,133]. Therefore, the future use of **7c** in combination with other kinase inhibitors to address its clinical resistance is possible. In the future, further structural optimization of existing ATP-competitive inhibitors to obtain safe, efficient, and potent RAF inhibitors can still be continued. Meanwhile, the development of allosteric inhibitors of RAF is also a promising area of interest.

### 2.8. Phosphatidylinositol 3 Kinases (PI3Ks) Inhibitors

**Figure 8:**
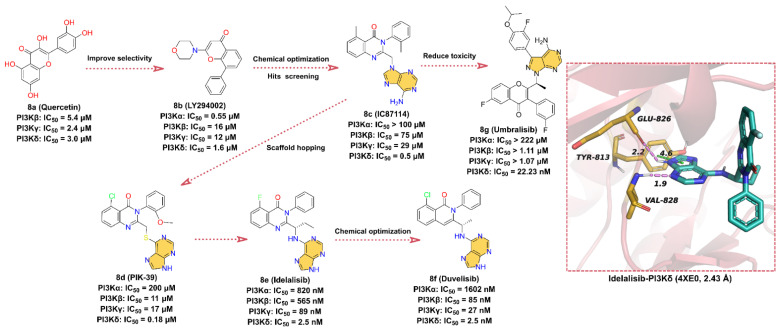
Key medicinal chemistry optimization of **PI3K** inhibitors along with the binding models with **PI3K**.

Human cells express three classes of phosphatidylinositol 3 kinases (PI3Ks), class I, II, and III, of which class I is the most widely studied and mainly includes PI3Kα, PI3Kβ, PI3Kγ, and PI3Kδ [134]. PI3Kα and PI3Kβ are commonly expressed, PI3Kδ and PI3Kγ are mainly expressed by leukocytes, and the activation of PI3Kγ is driven by activation of the G protein-coupled receptor (GPCR) with a more extensive expression pattern than PI3Kδ [135,136]. PI3Kδ has an impact on both the proliferation and function of B cells, providing ideas for the treatment of B cell-mediated malignancies [137]. PI3Ks are overexpressed in many types of tumor tissues, such as breast cancer, NSCLC, and colorectal cancer, and the development of PI3Ks inhibitors could be an effective strategy for tumor therapy [138]. PI3Ks inhibitors mainly cover pan-PI3K inhibitors and isoform-selective PI3K inhibitors [139]. The majority of pan-PI3K inhibitors are Pictilisib (GDC-0941), Buparlisib (BKM120), and Pilaralisib (XL147), while the major isoform-selective PI3K inhibitors include **Umbralisib**, **Duvelisib**, and **Idelalisib** [139,140,141,142]. In the following text, we focus on three isoform-selective PI3Ks inhibitors with azaindole cores (Figure 8).

In the early 1990s, Eli Lilly and Company obtained the PI3K inhibitor **8a** (**quercetin**) with IC_50_ values of 5.4, 2.4, and 3.0 μM for PI3Kβ, γ, and δ isoform, respectively, by screening a chemical library [143]. In order to improve the selectivity of **8a**, more druggable substituents are used to substitute catechol moiety, and the ATP-competitive PI3Ks inhibitor **8b** (**LY294002**) is finally obtained with IC_50_ values of 0.55, 16, 12, and 1.6 μM for PI3Kα, β, γ, and δ isoform, respectively. The aromatic ring linked to the morpholine scaffold in **8b** is embedded as a core pharmacophore in many PI3K inhibitors and is one of the most widely used tool compounds in biological research. In 2003, scientists from ICOS discovered **8c** (**IC87114**), an inhibitor that selectively inhibits PI3Kδ with an IC_50_ of 0.5 μM, by screening the SARs-optimized chemical library. Later, structural optimization of **8c** by scaffold hopping approach was performed to obtain **8d** (**PIK-39**). A comprehensive analysis of the crystal structure of **8d** complexed with PI3Kγ revealed that the binding conformation of this compound is distinct from the flat orientation of other PI3K inhibitors [144]. The isoquinolone moiety of **8d** projects upward to the roof of the ATP-binding pocket, and the kinase undergoes a conformational rearrangement in order to adapt to the inhibitor, where Met 804 shifts from an “up” position to a “down” position and forms an inducible drug-binding pocket. It is the unique binding model of **8d** to PI3K kinase that makes **8d** more selective for PI3Kδ kinase compared to other isoforms (IC_50_ = 0.18 μM) [136,143,144]. No detailed report of the development history of **8e** (**Idelalisib**) is available to date, but it binds in a similar conformation to **8d** at the ATP-binding site of the catalytic subunit of PI3Kδ [136], derived from **8c** [145,146]. Therefore, we presume that **8e** is designed based on the structure of **8d**. **8e** is an orally available, highly selective, first-in-class PI3Kδ inhibitor with an IC_50_ of 2.5 nM for the inhibition of p110δ, the catalytic subunit of PI3Kδ, and exhibits 40- to 300-fold selectivity for p110δ than for p110α/β/γ [147,148]. **8e** was approved by the FDA in July 2014 for the treatment of relapsed chronic lymphocytic leukemia (CLL), relapsed follicular B-cell non-Hodgkin lymphoma (NHL), and relapsed small lymphocytic leukemia (SLL) [140]. The cocrystal structure of **8e** with p110δ suggests that **8e** has a similar binding orientation to **8d**. The purine scaffold forms hydrogen bond interactions with key residues GLU826 and VAL828 in the hinge region of the ATP-binding site at distances of 2.2 and 1.9 Å, respectively. Moreover, the imidazole group of the purine scaffold can also form face-to-edge pi–pi interactions with residue TYR813.

**8f** (**Duvelisib**) is an important dual PI3Kδ/γ kinase inhibitor developed by Verastem that inhibits the PI3K catalytic subunits p110α, p110β, p110δ, and p110γ with IC_50_ values of 1602, 85, 2.5, and 27 nM, respectively [142,149]. In September 2018, the FDA approved **8f** for the treatment of patients with CLL/SLL or relapsed/refractory follicular lymphoma (FL) [142]. **8f** is structurally similar to **8e** and is also an ATP-competitive inhibitor discovered through a structure-based optimization approach [84,150,151,152]. The -F on **8e**’s isoquinolone is substituted with -Cl and the ethyl group is reduced to methyl to obtain the dual potent inhibitor of PI3Kδ/γ **8f**. Although no crystal structure of **8f** with PI3K has been reported, we suppose that **8f** shares the same binding pattern as **8e** and that the purine scaffold can form hydrogen bond interactions with key residues in the hinge region of the ATP-binding site. Additionally, the binding orientation of **8f** is similar to that of **8d**.

**8g** (**Umbralisib**) is a novel, orally available, selective, next-generation PI3Kδ inhibitor developed by TG Therapeutics with an IC_50_ of 22.23 nM [141,153]. **8g** was approved by the FDA in February 2021 for the treatment of several B-cell malignancies, including FL and relapsed/refractory marginal zone lymphoma (MZL) [141]. **8g** is structurally distinct from other PI3Kδ inhibitors. Based on the timeline of the drug discovery and the characteristics of the structure, we can infer that **8g** may also be optimized from **8c**. Since the N atom in the isoquinolone scaffold causes hepatotoxicity, the N is replaced by C or O atoms, which alters the toxicity distribution of **8g** [154]. Although the binding model of **8g** to PI3Kδ has not been released, the purine moiety is predicted to bind in the hinge region of the ATP-binding site and makes contact with key hinge residues based on its structural features. The 4-benzopyrone takes a similar orientation to **8e**, extending to the roof of the ATP-binding pocket, while the newly introduced 3-fluoro-4-isopropoxyphenyl binds to the internal hydrophobic pocket.

### 2.9. Tropomyosin-Related Kinases (TRK) Inhibitors

Neurotrophins binding to tropomyosin-related kinases (TRK) induce receptor dimerization, phosphorylation, and activation of downstream signaling cascades through PI3K, RAS/MAPK/ERK, and plc-γ pathways, which are associated with cell proliferation, differentiation, apoptosis, and survival of neurons and other cell types [155]. The TRK family includes TRKA, TRKB, and TRKC proteins, encoded by the neurotrophic receptor tyrosine kinase 1 (NTRK1), NTRK2, and NTRK3 genes, respectively, and these NTRK gene fusions are oncogenic drivers of various adult and pediatric tumor types [156,157]. The first-generation TRK inhibitors **Larotrectinib** and **Entrectinib** were approved by the FDA for the treatment of TRK fusion-positive cancers in November 2018 and August 2019, respectively [158,159,160]. In comparison, second-generation TRK inhibitors such as Selitrectinib and Repotrectinib are being investigated in clinical trials with the aim of addressing resistance to TRK mutations [161].

**Figure 9:**
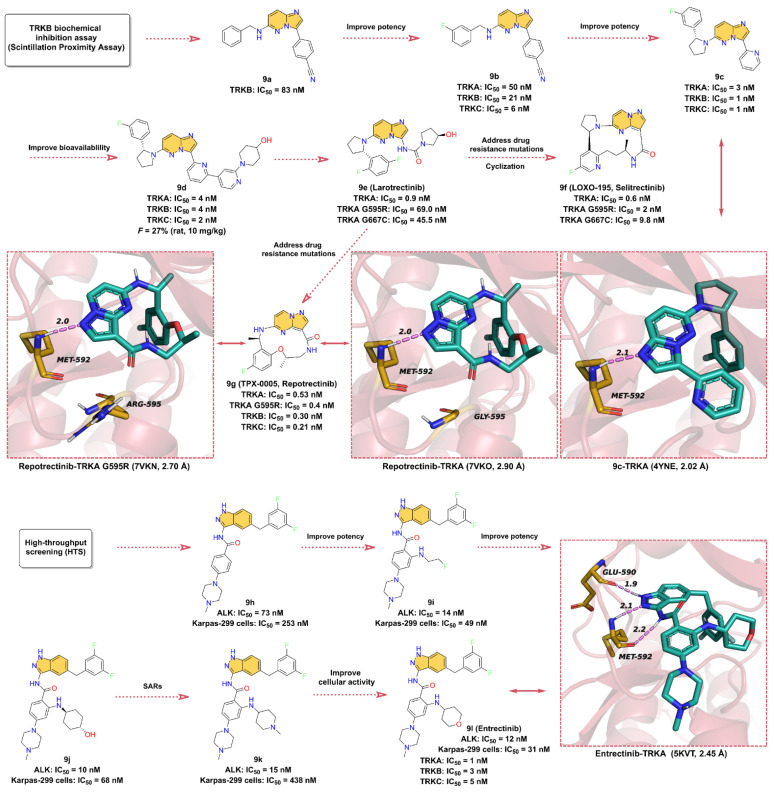
Key medicinal chemistry optimization of **TRK** inhibitors together with the binding models with **TRK**.

A TRKB inhibitor with novel benzonitrile-substituted imidazopyrazine **9a** is screened by a biochemical inhibition assay (Scintillation Proximity Assay) with an IC_50_ of 83 nM (Figure 9). The structure of **9a** is used as a starting point for further optimization of the TRK inhibitor [162]. In order to obtain inhibitors with better potency and PK properties, the electron-withdrawing groups such as -F is introduced at the 3-position of the phenylbenzylamine to obtain **9b**, which inhibits TRKA, TRKB, and TRKC with IC_50_ of 50, 21 nM, and 6 nM, respectively. Surprisingly, the rigidification of the phenylbenzylamine fraction by reducing the conformational entropy in a cyclic manner favors enhanced potency, with five-membered heterocyclic and *R*-enantiomeric showing the best performance. The **9c** with better potency is then obtained by replacing the 3-cyano-phenyl group with 2-pyridine moiety, and the IC_50_ for the inhibition of TRKA, TRKB, and TRKC are 3, 1, and 1 nM, respectively. According to the X-ray crystal structure, **9c** acts as an ATP-competitive inhibitor and binds to the ATP-binding site of TRKA. The -N on the imidazole of the imidazolopyrazine scaffold forms a hydrogen bond interaction with the hinge residue MET592 at a distance of 2.1 Å. The 3-fluorophenyl ring is bound in the hydrophobic region of the active site, while the pyridine group is in the solvent-exposed region. Further modification of the pyridine moiety in the solvent region leads to **9d**, which inhibits TRKA, TRKB, and TRKC with IC_50_ of 4, 4, and 2 nM, respectively. **9d** is further used for in vivo pharmacodynamic studies due to its pan-TRK potency, relatively low brain exposure, and overall acceptable PK properties (*F* = 27%) in vivo [162]. **9e** (**Larotrectinib**) is a highly selective TRK inhibitor developed by Loxo Oncology in collaboration with Bayer AG for the treatment of adult and pediatric patients with NTRK gene fusion-positive cancers, with IC_50_ of 1–20 nM for inhibition of TRKA, B, and C [159,163]. Based on the structural features, we speculate that **9e** was further optimized using **9d** as the lead compound. The problem of TRK resistance mutations has increasingly occurred in subsequent clinical practice. To overcome the challenge of acquired resistance, researchers have developed next-generation TRK inhibitors, of which **9f** (**Selitrectinib**, **LOXO-195**) and **9g** (**Repotrectinib**, **TPX-0005**) are two representatives [164]. Rational macrocyclization is an effective way to improve the activity of the compound on the target. Not only does it result in a relative reduction in molecular weight, but the increased rigidity of the structure also improves the potential for membrane permeability. **9f** and **9g** are optimized by macrocyclization based on the structure of **9e**. Using the cocrystal structure of **9g** and RTKA as an example, the -N on the pyrazole ring of the pyrimidinopyrazole scaffold still forms a hinge hydrogen bond with MET592 at a distance of 2.0 Å. When residue G595 of TRKA is mutated to G595R, the hinge hydrogen bond remains as expected, but the macrocyclized **9g** does not clash in space with the mutated G595R, indicating that the cyclized molecule is able to overcome the acquired drug resistance mutation of RTK.

**9l** (**Entrectinib**, Figure 9) is a potent, orally available, brain penetrant RTK inhibitor that targets TRKA, TRKB, TRKC, ROS1 (c-ros Oncogene 1 Kinase), and ALK (Anaplastic lymphoma kinase) with IC_50_ values of 1, 3, 5, 7, and 12 nM, respectively [165,166]. Interestingly, the development process of **9l** initially started with the target of ALK kinase [167,168]. The researchers use a high-throughput screening experiment to obtain compound **9h**, a 3-amino-5-substituted indazole derivative, which shows good inhibitory activity against ALK with an IC_50_ of 73 nM and also exhibits promising anti-proliferative activity against the ALK-dependent ALCL Karpas-299 cell lines with an IC_50_ of 253 nM. Optimization of the benzene ring attached to methylpiperazine using different substituents to obtain compound **9i**, which shows a significant increase in the inhibitory potency against ALK with an IC_50_ of 14 nM, as well as a remarkably elevated proliferation inhibitory activity against the Karpas-299 cells with IC_50_ of 49 nM. Further substitution of **9i** with 4-amino-4-cyclohexanol to afford **9j**, which further enhances its inhibitory potency on ALK (IC_50_ = 10 nM). In addition, **9k** and **9l** are obtained using 4-amino-*N*-methylpiperidinyl and 4-aminotetrahydropyranyl substitutions, respectively, both with good biochemical potency, and their IC_50_ values for ALK inhibition are 15 nM and 12 nM, respectively. However, **9k** exhibits (Karpas-299 cells, IC_50_ = 438 nM) worse cellular activity than **9l** (Karpas-299 cells, IC_50_ = 31 nM), which might be due to the low cell permeability. As mentioned earlier, **9l** is also a potent inhibitor of TRKs. The crystallographic structure reveals that **9l** binds in the ATP-binding pocket of TRKA and contacts with key residues around the binding site. In particular, the three N atoms on 3-aminoindazole scaffold can serve as hydrogen bond donors and acceptors to form three hydrogen bond interactions with the hinge residues GLU590 and MET592 at distances of 1.9, 2.1, and 2.2 Å, respectively.

### 2.10. Vascular Endothelial Growth Factor Receptors (VEGFRs) Inhibitors

**Figure 10:**
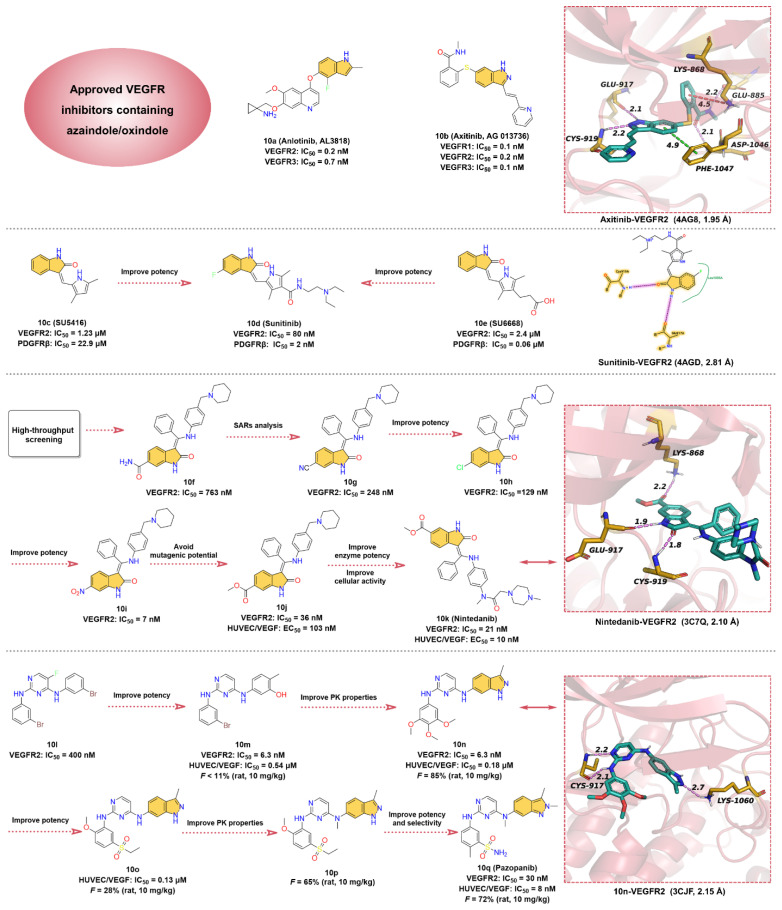
Key medicinal chemistry optimization of **VEGFR** inhibitors along with the binding models with **VEGFR2**.

Tumor angiogenesis is a prerequisite for tumor growth and metastasis [169], which is regulated by a number of pro- and anti-angiogenic factors produced by the host or tumor cells, including vascular endothelial growth factor (VEGF) and other cytokines [170,171]. Vascular endothelial growth factor receptors (VEGFRs) are members of the RTKs family and are classified into three isoforms, VEGFR1, VEGFR2, and VEGFR3 [172]. VEGFR1 and VEGFR2 play important roles in angiogenesis, including tumor angiogenesis, while VEGFR3 is associated with lymphangiogenesis [173]. Compared to normal endothelial cells, VEGFR2 activation by VEGF triggers a phosphorylation process that leads to greatly enhanced proliferation and migration of endothelial cells [173,174]. EGFR2 is often overexpressed in tumor endothelial cells, so treatment of tumors can be achieved by inhibiting angiogenesis [175]. Selective inhibition of VEGFR kinases can inhibit angiogenesis and is currently a very successful clinical strategy in cancer therapy [176,177]. VEGFR kinase inhibitors are mainly classified into two types according to the binding model. Type I kinase inhibitors compete directly with ATP for binding at the ATP-binding site in the active form of the kinase, while type II kinase inhibitors bind to the adenine-binding and an additional hydrophobic back pocket at the ATP-binding site in the inactive form. More than 10 small-molecule VEGFR2 kinase inhibitors have been approved for various cancer therapies [176]. Here, we only discuss VEGFR2 inhibitors that contain the core structure of azaindole or oxindole, including the type I inhibitors **Anlotinib**, **Sunitinib**, **Nintedanib**, and **Pazopanib**, and the type II inhibitor **Axitinib** (Figure 10).

Quinoline and indole fragments have a variety of biological activities and play a crucial role in the development of anticancer drugs. The VEGFR2 kinase inhibitor **10a** (**Anlotinib**, **AL3818**) is one such example [178], which exhibits potent inhibition of VEGFR2 and VEGFR3 with IC_50_ values of 0.2 and 0.7 nM, respectively. **10a**, co-developed by Jiangsu Chia-Tai Tianqing Pharmaceutical and Advenchen Laboratories, was approved in 2018 by NMPA for the treatment of patients with locally advanced or metastatic NSCLC [179,180]. Preclinical studies have shown that **10a** inhibits VEGF/PDGF-BB/FGF-2-induced cell migration, angiogenesis, and capillary-like tube formation in endothelial cells and has a broad inhibitory effect on tumor angiogenesis and growth [181,182]. To date, no cocrystal structure of **10a** with VEGFR2 has been reported, but a molecular docking study has shown that **10a** can bind to the ATP-binding pocket of VEGFR2 [183]. The hinge residues GLU917 and CYS919 can form hydrogen bond interactions with the quinoline fragment of **10a**, while the indole moiety is located deep in the hydrophobic region of the ATP-binding pocket. Thus, we conclude that **10a** is a type I VEGFR inhibitor.

Renal cell carcinoma (RCC) is the most common form of kidney cancer. More than 200,000 patients are diagnosed with this disease worldwide each year, and approximately 100,000 deaths occur each year [184,185]. Starting with the pyrazole-styryl derivative, researchers obtained the small-molecule indazole derivative **10b** (**Axitinib, AG-013736**), a type II inhibitor of VEGFR kinases, using a truncated lead compound strategy and the introduction of a conformational constraint strategy for rational structure-based drug design [186,187]. **10b** is a potent and selective inhibitor of VEGFR1, 2, and 3 with IC_50_ values of 0.1, 0.2, and 0.1 nM, respectively [188]. **10b** is a highly potent and selective drug compared to other approved RCC active agents and is currently approved for the treatment of RCC [189,190]. The cocrystal structure reveals that the indazole moiety of **10b** forms two hydrogen bonds with the hinge residues GLU917 and CYS919 at distances of 2.1 and 2.2 Å, respectively. Moreover, the indazole core also establishes a pi–pi stacking interaction with the residue PHE1047. While the *N*-methylbenzamide motif extends deep into the back hydrophobic pocket of the ATP-binding site, forming hydrogen bonds and pi–cation interactions with residues ASP1046, GLU885, and LYS868, respectively. Additionally, the pyridine-vinyl part of **10b** is in the hinge region and forms van der Waals interactions with surrounding residues.

**10d** (**Sunitinib**), a multi-target RTK inhibitor, was FDA-approved for the treatment of RCC, gastrointestinal stromal tumors, and progressive neuroendocrine tumors of pancreatic origin in 2006 [191,192]. Researchers have conducted extensive medicinal chemistry investigations on existing selective VEGFR2 inhibitors and selective platelet-derived growth factor receptor β (PDGFRβ) inhibitors in an effort to obtain potent antitumor drugs that can inhibit both VEGFR2 and PDGFRβ [193]. **10c** (**SU5416**) is a selective VEGFR2 inhibitor with an IC_50_ of 1.23 μM for VEGFR2 and 22.9 μM for PDGFRβ. **10e** (**SU6668**) is a selective PDGFRβ inhibitor with an IC_50_ of 0.06 μM for PDGFRβ and 2.4 μM for VEGFR2. Based on the structures of **10c** and **10e**, the researchers modified the C-4 position on the pyrrole ring to obtain **10d**. The SAR implied that **10d** exhibited potent inhibitory activity against both VEGFR2 and PDGFRβ with IC_50_ values of 80 and 2 nM, respectively. The crystal structure of VEGRF2 in complex with **10d** reveals that the indolin-2-one core forms two hinge hydrogen bonds with residues GLU917 and CYS919, which further anchor **10d** at the ATP-binding pocket.

**10k** (**Nintedanib**), a potent triple angiokinase (VEGFR/PDGFR/FGFR) inhibitor, is approved by FDA to treat idiopathic pulmonary fibrosis by blocking fibroblast proliferation and reducing extracellular matrix deposition [194,195]. In particular, **10k** has the most potent kinase inhibitory effect on VEGFR2 with an IC_50_ of 21 nM [196]. The researchers obtained the hit compound **10f** against VEGFR2 with an IC_50_ of 763 nM through high-throughput screening methods for selective testing of derivatives of related kinases [197]. It was found that the vertical conformation of the central benzene ring and the benzopyrrole scaffold can increase the solubility of the compound, so these key fragments are retained in the subsequent structure optimization process [194]. SARs studies on **10f** produced a more active compound **10g,** with an IC_50_ of 248 nM against VEGFR2 [195]. After replacing the -CN group on benzopyrrole with -Cl, the potency of compound **10h** was further enhanced with an IC_50_ of 129 nM. Interestingly, the -NO_2_ moiety has the strongest inhibitory effect on VEGFR2 with an IC_50_ of 7 nM (**10i**) but is eventually terminated due to a mutagenic potential. Despite the risk of degradation by esterase metabolism by replacing the -NO_2_ group with -COOCH_3_, **10j** yields an effective inhibition for VEGFR2 with an IC_50_ of 36 nM and moderate cytotoxicity (HUVEC/VEGF: EC_50_ = 103 nM). In addition, the piperidine fragment is optimized to fine-tune properties such as cellular activity and solubility, resulting in **10k** (HUVEC/VEGF: EC50 = 10 nM), a potent and selective VEGFR2 inhibitor [197]. Based on the X-ray cocrystal structure, it can be concluded that **10k** binds into the ATP-binding site of VEGFR2. The -NH and -CO groups on the indolinone scaffold can form two strong hydrogen bonds with the backbone carbonyl oxygen of GLU917 and the backbone nitrogen of CYS919 in the hinge region at distances of 1.9 and 1.8 Å, respectively. The phenyl group binds in the hydrophobic pocket of the ATP-binding site, forming hydrophobic interactions with the surrounding residues, while *N*-methylpiperazine group points into the solvent region of the active site, forming ionic and polar interactions.

**10q** (**Pazopanib**) is an oral multi-kinases inhibitor that primarily inhibits the VEGFRs with IC_50_ of 10, 30, and 47 nM for VEGFR1, 2, and 3, respectively, and is currently approved for the treatment of advanced soft-tissue sarcoma (STS) and RCC [198,199]. Compound **10l** with an inhibitory effect on VEGFR2 (IC_50_ = 400 nM) is obtained as a hit compound by screening the compound library [199]. Substitution of -Br in **10l** using -OH group gives **10m**, which inhibits VEGFR2 with an IC_50_ of 6.3 nM. It is presumed that the reason for this hundred-fold increase in activity may be attributed to the newly introduced -OH can form hydrogen bond interactions with key residues in the binding pocket [199]. Replacement of the benzene ring of **10m** with a 3-methylindazole heterocycle affords **10n**, which has significantly improved PK properties. The clearance of **10n** in rats was 16 mL/min/kg at an administered dose of 10 mg/kg, with an oral bioavailability of 85%. The cocrystal structure of VEGFR2 in complex with **10n** demonstrates that the 3-methylindazole motif can form a hydrogen bond with residue LYS1060 at a distance of 2.7 Å, and the 2-aminopyrimidine segment can form bidentate hydrogen bonds with the hinge residue CYS917 at distances of 2.1 and 2.2 Å, respectively (Figure 10, lower right). To further enhance the potency, the introduction of 5-sulfone in aniline to obtain **10o** leads to a moderate increase in the potency of the enzyme and cellular assay with IC_50_ of 130 nM in human umbilical vein endothelial cells (HUVEC). Methylation of the C-4 amino of the pyrimidine group to obtain **10p** can significantly improve the PK properties. To reduce the risk of nitrogen on the indazole heterocycle binding to heme iron of cytochrome P450 enzymes, its methylation to increase the steric resistance of the heterocycle and also optimization of the substituents on the aniline yields **10q** with desirable selectivity and cellular potency with an IC_50_ of 30 nM against VEGFR2 and 8 nM against HUVEC/VEGFR.

### 2.11. Others

**Figure 11:**
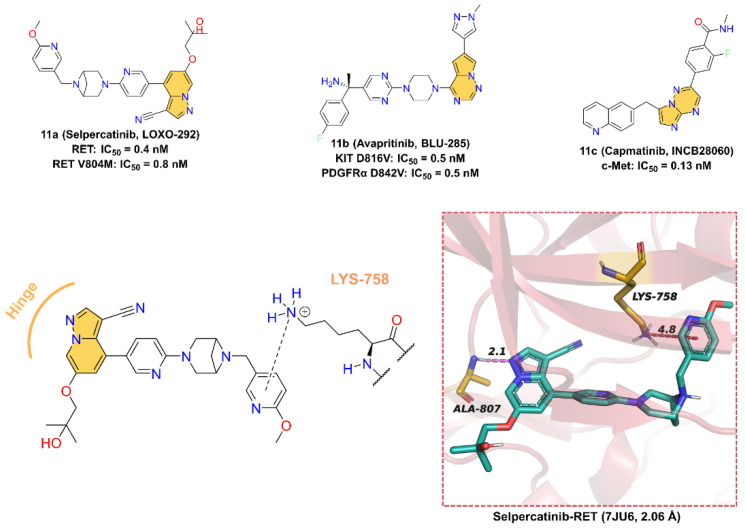
Two-dimensional structures of **Selpercatinib**, **Avapritinib**, and **Capmatinib** together with the binding pattern of **Selpercatinib** and RET.

Additionally, there are several other ATP-competitive small-molecule inhibitors containing azaindoles that have been approved in clinical, including **Selpercatinib**, **Avapritinib**, and **Capmatinib** (Figure 11).

**11a** (**Selpercatinib**, **LOXO-292**), developed by Loxo Oncology for the treatment of various solid tumors, including NSCLC and thyroid cancer, is a highly selective receptor tyrosine kinase rearranged during transfection (RET) inhibitor with IC_50_ values of 0.4 nM and 0.8nM for RET wild-type and mutant-type (V804M), respectively [200,201]. The cocrystal structure of RET in a complex with **11a** reveals that **11a** binds at the ATP-binding site. The pyrazolo [1,5-*a*]pyridine scaffold forms a hinge hydrogen bond with the backbone -NH of residue ALA807 at a distance of 2.1 Å, and the 2-methoxypyridine group inserts into the deep hydrophobic pocket forming a pi–cation interaction with residue LYS758.

**11b** (**Avapritinib**, **BLU-285**), developed by Blueprint Medicines for the treatment of gastrointestinal stromal tumors and systemic mastocytosis, is a potent, selective, orally active inhibitor of KIT and PDGFRα activation loop mutant kinases with IC_50_ values of 0.5 and 0.5 nM for KIT D816V and PDGFRα D842V mutant, respectively [202,203]. A molecular docking study was used to investigate the potential binding interactions between **11b** and KIT D816V. According to the results, **11b** binds at the ATP-binding site, and the pyrrolo [2,1-*f*][1,2,4]triazine scaffold makes one hinge hydrogen bond with the backbone of residue CYS673 at a distance of 2.4 Å [204].

**11c** (**Capmatinib**, **INCB28060**) is a potent, highly selective, ATP-competitive, and reversible c-Met kinase inhibitor with an average IC_50_ value of 0.13 nM [205]. On May 6, 2020, **11c** received its first worldwide U.S. approval for the treatment of adults with metastatic NSCLS [206,207,208,209]. **11c** was first reported by patent (US8420645B2) as a c-MET kinase inhibitor with an imidazo [1,2-*b*][1,2,4]triazin scaffold. However, the crystal structure of **11c** with c-Met has not been disclosed so far, and therefore, the binding model to the protein cannot be accurately determined. Nevertheless, we can speculate that the imidazolotriazine core is an indispensable pharmacophore for binding to the ATP-binding site of c-Met.

## 3. Conclusions

Over the past 20 years, protein kinases have been attracting great interest, and significant achievements have already been made in developing ATP-competitive kinase inhibitors. A variety of kinase inhibitors investigated in preclinical and clinical studies have indole/azaindole/oxindole scaffolds. In particular, thirty indole/azaindole/oxindole-containing ATP-competitive kinase inhibitors have been approved as therapeutic agents for various diseases. This paper describes the discovery and optimization process of these thirty approved, another three in clinical trials, an additional one in preclinical studied indole/azaindole/oxindole-based ATP-competitive kinase inhibitors, including Bcr-Abl, BTK, CDK4/6, CSF1R, HER, JAK, BRAF, PI3K, TRK, VEGFR, and other kinase inhibitors (Table 1). Meanwhile, the drug mutant routes of these selected drugs were summarized to verify the potential contribution of indole-like structural fragments to their overall biological activity and therapeutic effect. We also explore the interactive bonding between the inhibitors and amino acid residues at the APT-binding sites of their target kinase based on the corresponding protein crystal complex. It is found that indole/azaindole/oxindole scaffolds can form hinge hydrogen bonds and other non-covalent bond interactions with residues in the hinge region of the kinase’s APT-binding site to better bind to the ATP-binding pocket. Convincingly, indole/azaindole/oxindole structures play an indispensable role in occupying the ATP pocket. This implies that these heterocyclic systems are a privileged scaffold in the quest for new ATP-competitive kinase inhibitors.

At present, kinase inhibitors remain the first choice for many diseases. However, mutations in the residues of the kinase hinge region generally lead to drug resistance that reduces the potency and selectivity of the inhibitor. Additionally, the period from R&D to market for small-molecule inhibitors is long, and the investment is enormous. Therefore, the authors hope this review will help researchers quickly develop more selective and potent indole/azaindole/oxindole-based kinase inhibitors combined with artificial intelligence technology.

## Figures and Tables

**Table 1:** Indole/azaindole/oxindole-containing ATP-competitive kinase inhibitors in clinical applications.

No.	INN	Company	Indications	Approval/Clinical Trial No.	Patients
1e	Ponatinib	Ariad, Cambridge, MA, USA	hematological cancers: chronic myeloid leukemia (CML): T315 resistant	FDA (2012) [210]	patients with CML or Philadelphia chromosome-positive (Ph1) acute lymphoblastic leukemia (ALL) that is resistant to or intolerant of prior TKI therapy
2b	Ibrutinib	Pharmacyclics Inc., Sunnyvale, CA, USA	hematological cancers: mantle cell lymphoma (MCL) chronic lymphocytic leukemia/small lymphocytic lymphoma (CLL/SLL)	FDA (2013) [211]	previously treated patients with MCL
2c	Acalabrutinib	AstraZeneca, Cambridge, UK	hematological cancers: mantle cell lymphoma	FDA (2017) [212]	patients with relapsed/refractory MCL
2d	Tirabrutinib	Ono, Osaka, Japan	hematological cancers: recurrent or refractory primary central nervous system lymphoma(R/R PCNSL)	Japan (2020) [213]	patients with PCNSL
2f	Nemtabrutinib	Merck Sharp & Dohme LLC, Rahway, NJ, USA	hematological cancers: chronic lymphocytic leukemia/small lymphocytic lymphoma	Phase 3 NCT05624554	Chronic lymphocytic leukemia/small lymphocytic leukemia
3d	Abemaciclib	Eli Lilly, Indianapolis, IN, USA	solid tumor: hormone receptor (HR)-positive, human epidermal growth factor receptor 2 (HER2)-negative advanced or metastatic breast cancer	FDA (2017) [214]	patients with HR-positive, HER2-negative advanced or metastatic breast cancer
3j	Ribociclib	Novartis, Basel, Switzerland	solid tumor: advanced breast cancer	FDA (2017) [215]	post-menopausal women with hormone receptor-positive, human epidermal growth factor receptor 2-negative advanced or metastatic breast cancer
4e	Pexidartinib	Daiichi Sankyo, Tokyo, Japan	solid tumor: tenosynovial giant cell tumor	FDA (2019) [216]	adult patients with symptomatic TGCT associated with severe morbidity or functional limitations and not amenable to surgery
5f	Tucatinib	Seattle Genetics, Bothell, WA, USA	solid tumor: advanced unresectable or metastatic HER2-positive breast cancer	FDA (2020) [217]	adult patients with advanced, unresectable, or metastatic HER2-positive breast cancer, including patients with brain metastases, who received one or more prior anti-HER2-based regimens in the metastatic setting
5g	Osimertinib	AstraZeneca, Cambridge, UK	solid tumor: metastatic EGFR T790M mutation-positive non-small-cell lung cancer	FDA (2015) [88]	patients with metastatic EGFR T790M mutation-positive non-small-cell lung cancer who have progressed on or after EGFR TKI therapy
5h	Almonertinib	Hansoh, Lianyungng, Jiangsu	solid tumor: advanced EGFR T790M + non-small-cell lung cancer	NMPA (2020) [218]	advanced and metastatic NSCLC patients harboring sensitive EGFR or T790 M mutation
6a	Ruxolitinib	Incyte Corporation, Wilmington, DE	myeloproliferative neoplasms: myelofibrosis (MF) hydroxyurea(HU)-resistant or -intolerant polycythemia vera (PV)	FDA (2011), EMA (2012) [219]	MF patients and PV patients
6b	Baricitinib	Eli Lilly/Incyte, Indianapolis, IN, USA/Wilmington, Delaware, USA	rheumatoid arthritis	FDA (2017) [105]	adult patients with rheumatoid arthritis
6f	Tofacitinib	Pfizer, Brooklyn, NY, USA	rheumatoid arthritis psoriatic arthritis ulcerative colitis polyarticular course juvenile idiopathic arthritis	FDA (2012) [220]	patients with moderate to severe rheumatoid arthritis (RA), psoriatic arthritis (PA), ulcerative colitis (UC), and polyarticular course juvenile idiopathic arthritis (pcJIA)
6g	Delgocitinib	Japan Tobacco Co., Toyko, Japan	atopic dermatitis	Japan (2020) [221]	adults with atopic dermatitis
6j	Peficitinib	Astellas Pharma, Toyko, Japan	rheumatoid arthritis	Japan (2019) [222]	patients who have an inadequate response to conventional therapies
6m	Filgotinib	Galapagos/Abbott, Chicago, IL, USA	rheumatoid arthritis	EMA (2020), Japan (2020) [223]	patients who had an inadequate response to conventional therapies
7c	Vemurafenib	Hoffmann La Roche, Basel, Switzerland	metastatic and unresectable melanoma with V600 mutation	FDA (2011) [224]	patients with malignant melanoma with BRAF V600E positive mutation
8e	Idelalisib	Gilead Sciences, Foster City, CA, USA	relapsed chronic lymphocytic leukemia	FDA (2014) [139]	patients for whom rituximab alone would be an appropriate therapy due to other co-morbidities
8f	Duvelisib	Verastem Oncology, Needham, MA	chronic lymphocytic leukemia/small lymphocytic lymphoma or relapsed/refractory follicular lymphoma (FL)	FDA (2018) [142]	adult patients with relapsed or refractory chronic lymphocytic leukemia (CLL)/small lymphocytic lymphoma (SLL) after at least two prior therapies patients with relapsed or refractory follicular lymphoma (FL) after at least two prior systemic therapies
8g	Umbralisib	TG Therapeutics, Morrisville, NC, USA	relapsed or refractory follicular lymphoma (FL) and relapsed/refractory marginal zone lymphoma (MZL)	FDA (2021) [141]	adults with relapsed or refractory marginal zone lymphoma (MZL) who have received ≥1 prior anti-CD20-based regimen and relapsed or refractory follicular lymphoma (FL) who have received ≥3 prior lines of systemic therapy
9e	Larotrectinib	Loxo Oncology, New York, NY, USA	NTRK gene fusion-positive cancers(non-small-cell lung cancer, thyroid, salivary gland, colorectal, biliary, primary CNS)	FDA (2018) [159]	adult and pediatric patients with solid tumors that have an NTRK gene fusion without a known acquired resistance mutation
9f	Selitrectinib	Bayer, Leverkusen, Germany	solid tumors (e.g., non-small-cell lung cancer, thyroid, salivary gland, colorectal, biliary, primary CNS) Harboring NTRK Fusion	Phase 1 NCT04275960	adult patients with cancer having a change in a particular gene (NTRK1, NTRK2, or NTRK3 gene fusion)
9g	Repotrectinib	Memorial Sloan Kettering Cancer Center, New York, NY, USA	advanced or metastatic EGFR mutant non-small-cell lung cancer	Phase 1 NCT04772235	patients with advanced or metastatic EGFR mutant non-small-cell lung cancer (NSCLC)
9l	Entrectinib	Genentech, South San Francisco, CA, USA	Solid tumors (e.g., breast cancer, cholangiocarcinoma, colorectal cancer, gynecological cancer, pancreatic cancer, and thyroid cancer) harboring NTRK1/2/3 or ROS1 gene fusions	Japan (2019) [160]	adult and pediatric patients with NTRK fusion-positive, advanced or recurrent solid tumors
10a	Anlotinib	Advenchen Laboratories, Moorpark, CA, USA	locally advanced or metastatic non-small-cell lung cancer	NMPA (2018) [179]	patients with locally advanced or metastatic non-small-cell lung cancer (NSCLC) who have undergone progression or recurrence after ≥2 lines of systemic chemotherapy
10b	Axitinib	Pfizer, Brooklyn, NY, USA	metastatic renal cell carcinoma	FDA (2012), EMA (2012) [225,226]	patients with metastatic renal cell carcinoma after failure of one prior systemic therapy
10d	Sunitinib	Pfizer, Brooklyn, NY, USA	advanced renal cell carcinomas gastrointestinal stromal tumors	FDA (2006) [227]	RCC patientsimatinib-resistant GIST patients
10k	Nintedanib	Boehringer Ingelheim, Ingelheim am Rhein, Germany	idiopathic pulmonary fibrosis	FDA (2014) [228]	patients with idiopathic pulmonary fibrosis
10q	Pazopanib	GSK, Brentford, UK	renal cell carcinoma advanced soft-tissue sarcoma	FDA (2009, 2012) [229]	patients with locally advanced unresectable or metastatic renal cell carcinoma (RCC) patients who have received prior chemotherapy, excluding those with adipocytic STS or gastrointestinal stromal tumor (GIST)
11a	Selpercatinib	Eli Lilly, Indianapolis, IN, USA	RET fusion-positive non-small-cell lung cancer, RET fusion-positive thyroid cancer, and RET-mutant medullary thyroid cancer	FDA (2020) [200]	adult patients with metastatic RET fusion-positive NSCLC, adult and pediatric patients ≥ 12 years of age with advanced or metastatic RET-mutant medullary thyroid cancer who require systemic therapy, and adult and pediatric patients ≥ 12 years of age with advanced or metastatic RET fusion-positive thyroid cancer who require systemic therapy and who are radioactive iodine-refractory (if radioactive iodine is appropriate)
11b	Avapritinib	Blueprint Medicines, Cambridge, MA, USA	unresectable or metastatic gastrointestinal stromal tumors harboring a PDGFRA exon 18 mutation, including PDGFRA D842V mutations	FDA (2020) [203]	adults with unresectable or metastatic gastrointestinal stromal tumors harboring a PDGFRA exon 18 mutation, including PDGFRA D842V mutations
11c	Capmatinib	Novartis, Basel, Switzer-land	metastatic non-small-cell lung cancer	FDA (2020) [206]	adults with metastatic non-small-cell lung cancer (NSCLC) whose tumors have a mutation that leads to MET exon 14 skipping

## Data Availability

Not applicable.
